# Prevention and management of foot and lower limb health complications in adults undergoing dialysis: a scoping review

**DOI:** 10.1186/s13047-023-00679-z

**Published:** 2023-11-20

**Authors:** Sarah M. Manewell, Purnima Rao, Keren Haneman, Minjia Zheng, Hady Charaf, Hylton B. Menz, Cathie Sherrington, Serene S. Paul

**Affiliations:** 1 School of Health Sciences, Faculty of Medicine and Health, The University of Sydney, NSW, Camperdown, Australia; 2 Podiatry Department, Sydney Local Health District, NSW Health, Camperdown, Australia; 3 School of Public Health, Faculty of Medicine and Health, The University of Sydney, NSW, Camperdown, Australia; 4 Faculty of Podiatric Medicine, School of Health Sciences, Western Sydney University, NSW, Campbelltown, Australia; 5 School of Allied Health, Human Services and Sport, La Trobe University, Melbourne, Victoria Australia; 6 Institute for Musculoskeletal Health, Faculty of Medicine and Health, The University of Sydney/Sydney Local Health District, NSW, Camperdown, Australia

**Keywords:** Dialysis, End stage renal disease, Foot, Lower limb

## Abstract

**Background:**

Foot and lower limb health complications are common among patients undergoing dialysis; but a summary of prevention and management evidence is not available. The aim of this scoping review was to summarise study characteristics and the nature of results regarding strategies to prevent and manage peripheral arterial disease (PAD), foot ulceration, amputation, associated infection and associated hospital admission in adults undergoing dialysis.

**Methods:**

MEDLINE, Embase, CINAHL and AMED databases were searched for longitudinal experimental and observational studies. Eligible studies included adults undergoing dialysis (≥10 dialysis patients, with separate results or ≥ 75% of the cohort). Any interventions relating to PAD, foot ulceration, amputation, associated infection, and associated hospital admission were included.

**Results:**

The review included 212 studies, of which 199 were observational (94%) and 13 were experimental (6%). Sixteen studies (8%) addressed the prevention of foot and lower limb health complications, 43 (20%) addressed management, and 153 (72%) addressed both. The main intervention type in each study was surgery (*n* = 159, 75%), care from one or more health professionals (*n* = 13, 6%), screening by a health professional (*n* = 10, 5%), medication (*n* = 9, 4%) and rehabilitation (*n* = 5, 2%). No studies were identified where exercise, offloading or education were the main intervention. Results for PAD were reported in 137 (65%) studies, foot ulceration in 54 (25%), amputation in 171 (81%), infection in 7 (3%), and admission in 26 studies (12%). Results for more than one foot or lower limb outcome were reported in 141 studies (67%), with each study reporting on average two outcomes. Results varied and spanned positive, negative, and neutral outcomes following intervention.

**Conclusions:**

Identified studies frequently aimed to both prevent and manage foot and lower limb health complications. A variety of interventions were identified and studies often reported results for more than one foot or lower limb health outcome. Findings from this review can be used to guide future research, with a goal to support improved patient outcomes.

**Supplementary Information:**

The online version contains supplementary material available at 10.1186/s13047-023-00679-z.

## Introduction

Foot and lower limb health complications including peripheral arterial disease (PAD), foot ulceration, lower limb amputation, associated infection, and associated requirements for hospital admission are common among adults undergoing long term dialysis treatment. A previous study among 450 dialysis patients in Australia identified the prevalence of PAD at 52%, current foot ulceration at 10% and previous amputation (minor or major) at 10% [1]. Infection susceptibility is higher for patients with renal disease [2, 3], and 33% of hospital admissions over a 30-month follow-up period among 150 dialysis patients in the United States of America were related to foot health [4]. Of added complexity, more than one foot or lower limb health complication is often experienced by dialysis patients (e.g. 34% of ulcers precede amputation) [4] and these complications are often interrelated (e.g. more complex foot ulcers are associated with more complex infections) [3].

The risks associated with developing foot ulceration and amputation among dialysis patients are well established, including history of foot ulceration (odds ratio [OR] 17.6 and 70.1, respectively) and PAD (OR 7.5 and 9.1, respectively) [5]. Previous systematic reviews regarding PAD management have identified worse outcomes for dialysis patients [6, 7] and highlighted the need for further interventional research among this cohort to support evidence-based best practice [6].

In view of the rates and risks for dialysis patients, clinicians ask the question ‘what can we do to best prevent and manage foot and lower limb health complications?’ As this is a broad topic, conducting a scoping review was deemed appropriate to provide a summary of available evidence and guide future research [8]. Accordingly, the aim of this scoping review was to summarise the available evidence regarding the prevention and management of foot and lower limb health complications (PAD, foot ulceration, amputation, associated infection and the associated requirement for hospital admission) for adults undergoing dialysis. The specific focus was to report study characteristics, and the nature of outcomes for identified prevention and management strategies.

## Materials and methods

The protocol for this project was originally published prospectively via Prospero (*registration: 91268*) as a planned systematic review, however, initial findings indicated that a scoping review was more appropriate. An updated protocol was published retrospectively with Open Science Framework (https://osf.io/yacp8/). This scoping review was planned, conducted and reported in alignment with the JBI Manual for Evidence Synthesis (scoping review chapter) [9] and the Preferred Reporting Items for Systematic Reviews and Meta-Analyses-Scoping Review extension (PRISMA-SR) [8].

MEDLINE, Embase, CINAHL and AMED databases were searched from inception through 01 June 2022 using the search strategies outlined in Additional file 1. Records were initially de-duplicated using Endnote (Clarivate Analytics, Philadelphia, PA, USA), before all records were imported into Covidence software (Melbourne, Victoria, Australia). Title and abstract screening, full text review and data extraction were completed by pairs of reviewers (SMM, PR, KH, MZ, HC, SSP, GM and MC). Consensus regarding any differences between the two reviewers was reached by discussion or assessment by a third reviewer.

In view of the broad topic under investigation and as preliminary investigation suggested limited experimental studies, both experimental and observational studies were included where data were available from two or more time-points. Eligible study *populations* included a minimum of 10 adults with end stage renal disease (ESRD) who were receiving haemodialysis or peritoneal dialysis. Also, to ensure that findings related primarily to dialysis patients, results specifically for the dialysis patients had to be reported, or if combined results were reported then dialysis patients had to represent ≥75% of the cohort. Patients with renal transplant may have been included in the study population along with dialysis patients. Any *interventions* relating to the prevention and management of foot and lower limb *outcomes* PAD, foot ulceration, amputation, associated infection, or the associated requirement for hospital admission were included, with or without *comparison* groups. Exclusion criteria included: interventions addressing the prevention or management of calciphylaxis, aortic complications or acute PAD; inability to retrieve the publication; conference abstracts; non-original research; and, non-English language publications.

The outcomes of interest (Table 1) were extracted from each publication and presented as number and percentage, average or narrative summary. Results were synthesised using Microsoft Excel and SAS Enterprise Guide v7.1 (SAS Institute Inc. NC, USA).

**Table 1:** Outcomes extracted from each study

**Study characteristics** *General* Year of publication Publication title Primary author Aim Design: experimental (non-randomised controlled trial, randomised control trial) or observational (pre-post study or interrupted time series, cross-sectional study, cohort study, case control study) Clinical setting of intervention: dialysis unit (hospital or satellite), other inpatient setting, other outpatient setting, rehabilitation setting, or other Country Duration of intervention or follow-up: mean, median or total Comparator group: yes, no *Population* Number of dialysis patients Proportion of dialysis versus non-dialysis patients included in the study: < 75%, ≥ 75% Type of dialysis: haemodialysis, peritoneal dialysis, both Patients with transplant included: yes, no Diabetes: yes, no, yes and no Sex: males (%) Age: years (mean or median) **Interventions and outcomes** *Interventions* Type: surgery, medication, care from health professionals, screening by health professionals, rehabilitation, exercise, offloading, education, or other Prevention, management, or both prevention and management (e.g. prevent amputation while managing PAD): number of studies *Outcomes* PAD: number of studies, narrative outcome summary Foot ulceration: number of studies, narrative outcome summary Amputation: number of studies, narrative outcome summary Infection: number of studies, narrative outcome summary Related hospital admission: number of studies, narrative outcome summary Studies which report more than one result category: yes, no Average number of result categories	

## Results

**Fig. 1:**
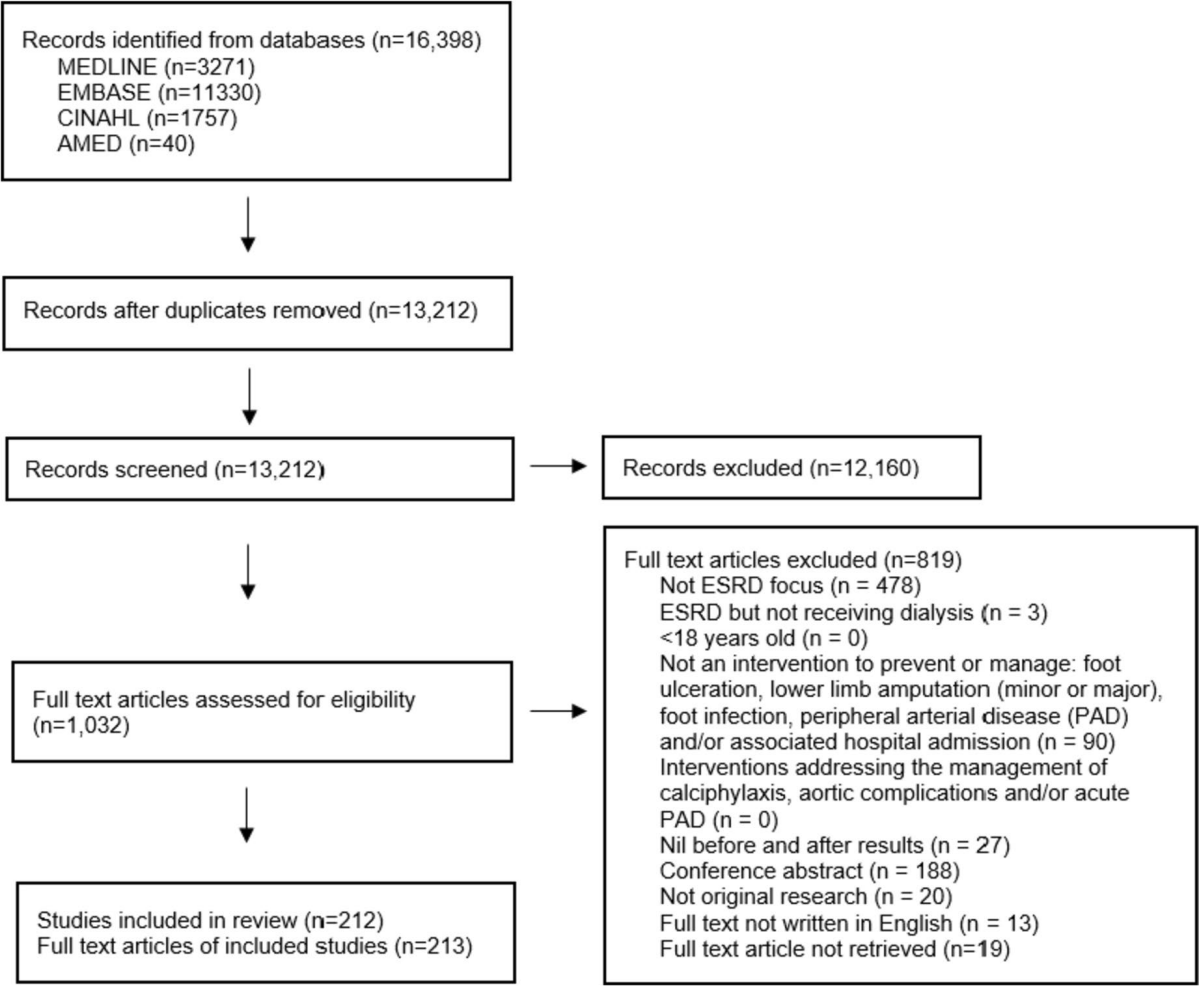
PRISMA-SR flow diagram of studies through the review

Database searches identified 16,398 records, of which 3186 duplicates were removed. Title and abstract screening were completed for 13,212 records, and full text screening for 1032 records. In total, 212 studies (in 213 reports) met the inclusion criteria and were included in this review (Fig. 1). See Additional file 2 for a table of extracted data and references of included studies.

### Study characteristics

Included reports were published between 1988 to 2022. Studies were primarily observational (*n* = 199, 94%), with only a small number of experimental studies identified (*n* = 13, 6%). The most common clinical setting where interventions were delivered, were inpatient and/or hospital settings other than dialysis units (*n* = 167, 79%). Studies were completed in 24 countries, the most common being the United States of America (*n* = 87, 41%), Japan (*n* = 55, 26%) and Germany (*n* = 10, 5%).

Duration of intervention or follow-up ranged from less than 6 months to 5 years or more, with the most common duration of intervention or follow-up being 1 year to less than 2 years (*n* = 66, 31%). Comparison groups were identified in 56 studies (26%).

The number of dialysis patients in each study ranged from 10 to 61,292. In 117 studies (55%), results were reported specifically for the dialysis patients, although in these studies dialysis patients comprised < 75% of the total study population. In the remaining 95 studies (45%), the dialysis patient population comprised ≥75% of the total study population. Studies most commonly investigated patients who underwent haemodialysis (*n* = 99, 47%). Additionally, studies most commonly investigated both patients with and without diabetes (*n* = 114, 54%). From all included studies, the weighted average age of included patients was 64 years and 66% were males. Additional study characteristic results are included in Table 2.

**Table 2:** Study characteristics

General	n	%
Experimental or observational		
Observational	199	94%
Experimental	13	6%
Study Design		
Cohort study	169	80%
Case series	14	7%
Case control study	12	6%
Before and after study or interrupted time series	4	2%
Cross sectional	0	0%
Non-randomised controlled study	9	4%
Randomised controlled trial	4	2%
Clinical setting of intervention		
Inpatient (hospital) setting (other than dialysis)	167	79%
Dialysis unit (hospital or satellite)	19	9%
Outpatient setting (other than dialysis)	10	5%
Rehabilitation unit	5	2%
Multiple settings	3	1%
Not reported	8	4%
Country		
United States of America	87	41%
Japan	55	26%
Germany	10	5%
Italy	9	4%
Canada	7	3%
Taiwan	6	3%
United Kingdom (England, Scotland, Wales, Northern Ireland)	5	2%
Finland	3	1%
Israel	3	1%
Singapore	3	1%
China	2	1%
Czech Republic	2	1%
France	2	1%
Greece	2	1%
Poland	2	1%
The Netherlands	2	1%
Australia	1	0.5%
Austria	1	0.5%
Korea	1	0.5%
Portugal	1	0.5%
Spain	1	0.5%
Sweden	1	0.5%
Switzerland	1	0.5%
Turkey	1	0.5%
Multiple countries	2	1%
Not reported	2	1%
Duration of intervention or follow-up		
Less than 6 months	21	10%
6 months to less than 1 year	11	5%
1 year to less than 2 years	66	31%
2 years to less than 5 years	55	26%
5 years or more	45	21%
Not reported	14	7%
Comparator group		
Yes	56	26%
No	156	74%
**Population**		
Proportion of dialysis versus non-dialysis patients included in the study		
< 75%	117	55%
≥75%	95	45%
Type of dialysis		
Haemodialysis	99	47%
Peritoneal dialysis	2	1%
Haemodialysis and peritoneal dialysis	43	20%
Not reported	68	32%
Renal transplant patients included		
Yes	31	15%
No	181	85%
Diabetes		
Yes	32	15%
No	0	0%
Yes and no	114	54%
Not reported	66	31%

### Interventions and outcomes

Most studies were interventions that aimed to both prevent and manage foot and lower limb health complications (*n* = 153, 72%). Fewer study interventions aimed to manage (*n* = 43, 20%) or prevent (*n* = 16, 8%) foot and lower limb health complications in isolation.

Surgery was the main intervention in 159 studies (75%), examples of which included endovascular and bypass revascularisation, minor and major amputation, and skin grafting. Care from one or more health professionals was the main intervention in 13 studies (6%), examples of which included patients attending a wound care centre, patients attending a multidisciplinary diabetes related foot clinic, and a specialist podiatrist attending dialysis to provide foot care. Screening by a health professional was the main intervention in 10 studies (5%), examples of which included skin perfusion pressure assessment, ankle-brachial and toe-brachial indices assessment and nurse led foot screening during dialysis. Medication was the main intervention in nine studies (4%), examples of which included vancomycin for the management of methicillin-resistant staphylococcus aureus infection and vitamin K antagonist therapy in the presence or absence of ulceration, PAD and amputation. Rehabilitation was the main intervention in five studies (2%), examples of which included staying at a rehabilitation unit following major amputation and undertaking physical therapy following minor amputation. No studies were identified where exercise, offloading or education were the main intervention. Other interventions were evident in 16 studies (8%), examples of which included low-density lipoprotein apheresis, negative pressure wound therapy and the introduction of treatment guidelines along with an outreach wound care clinic.

Results for more than one foot or lower limb health outcome (PAD, foot ulceration, amputation, related infection, and associated hospital admission) were reported in 141 studies (67%). These studies each reported an average of 2 foot and lower limb health outcomes. PAD results were present in 137 studies (65%). Examples of PAD related outcomes included patency, change in ankle brachial or toe pressure indices, change in pain, and progression to reintervention. Foot ulceration results were present in 54 studies (25%). Examples of foot ulcer related outcomes included healing rate and healing time. Amputation results were present in 171 studies (81%). Examples of amputation related outcomes included time to amputation and rates of ambulation post procedure. Infection results were present in 7 studies (3%). Examples of infection related outcome included rates of infection developing, resolving, and deteriorating. Hospital admission results were present in 26 studies (12%). Hospital admission related outcomes were primarily rate of admission and associated length of stay. Outcome results (positive, negative, or neutral) from included studies were variable. Results for foot and lower limb health outcomes according to intervention type, including consideration of whether studies aimed for prevention or management, are documented in Table 3.

**Table 3:** Study results by intervention type, prevention or management goals, and foot or lower limb health outcomes

Intervention	Prevention or management						Foot and lower limb health outcome									
	Prevention (*n* = 16)		Management (*n* = 43)		Prevention and management (*n* = 153)		PAD (*n* = 137)		Foot ulcer (*n* = 54)		Amputation (*n* = 171)		Infection (*n* = 7)		Foot and lower limb health related hospital admission (*n* = 26)	
	n	%	n	%	n	%	n	%	n	%	n	%	n	%	n	%
Surgery (*n* = 159)	1	6%	30	70%	128	84%	110	80%	36	67%	138	81%	4	67%	16	64%
Medication (*n* = 9)	5	31%	3	7%	1	1%	7	5%	1	2%	7	4%	1	17%	2	8%
Care from health professionals (*n* = 13)	2	13%	0	0%	11	7%	5	4%	5	9%	8	5%	1	17%	3	12%
Screening by health professionals (*n* = 10)	4	25%	1	2%	5	3%	5	4%	5	9%	6	4%	0	0%	0	0%
Rehabilitation (*n* = 5)	0	0%	4	9%	1	1%	0	0%	0	0%	5	3%	0	0%	3	12%
Exercise (*n* = 0)	0	0%	0	0%	0	0%	0	0%	0	0%	0	0%	0	0%	0	0%
Offloading (*n* = 0)	0	0%	0	0%	0	0%	0	0%	0	0%	0	0%	0	0%	0	0%
Education (*n* = 0)	0	0%	0	0%	0	0%	0	0%	0	0%	0	0%	0	0%	0	0%
Other (*n* = 16)	4	25%	5	12%	6	4%	10	7%	7	13%	6	4%	0	0%	1	4%

## Discussion

Most studies identified in this review aimed to both prevent and manage foot and lower limb health complications (*n* = 153, 72%). A common example were studies which investigated surgical management for PAD but also aimed to prevent amputation. This aligns with our finding that more than one foot or lower limb health outcome (PAD, foot ulceration, amputation, related infection, and associated hospital admission) was reported in 67% of identified studies (*n* = 141). As noted previously, often more than one foot or lower limb health complication is experienced by adults undergoing dialysis [4] and these complications are frequently interrelated [3]. As such, an interdisciplinary approach to both research and care is essential. It is positive that guidelines which promote interdisciplinary care for foot and lower limb health are acknowledging dialysis patients as a particularly high risk group [10, 11]. Of note, foot and lower limb health guidelines are primarily targeted to patients with diabetes [10, 11], however, in practice these guidelines are often applied to patients without diabetes who experience the same or similar complications. As dialysis patients without diabetes also experience foot and lower limb health complications [1], it is encouraging that patients without diabetes were included in 54% of studies (*n* = 114) identified in this review.

The outcome results (positive, negative, or neutral) identified in this review were variable. For example, one study aimed to manage major amputation via rehabilitation and reported an ambulatory rate of 74% for patients with below knee amputation, however, most patients experienced difficulties with prosthetic fit [12]. This required an average length of stay of 74 days, and during rehabilitation 43% of patients (*n* = 13) required transfer to an acute hospital unit [12], identifying results which were clinically positive, neutral and negative. A different study which also aimed to manage major amputation via rehabilitation reported that on discharge 26% of patients were independent, 26% achieved partial independence and 47% were dependent, requiring complete assistance [13], again demonstrating clinically variable results. These variable results related in part to the broad inclusion criteria employed in the current scoping review, including various foot and lower limb health outcomes (PAD, foot ulceration, etc.), interventions, overall management goals (prevention and/or management), and reported outcomes (arterial patency, foot ulcer healing rates, etc.). We believe that the benefits of completing this broad review (due to foot and lower limb health being complex and interrelated for dialysis patients) outweigh this limitation. Of note, some positive results were identified, such as reduced rates of major amputation with the introduction of routine foot screening (17% reduction for rate of major amputation, *p* = 0.003) [14].

Most studies identified in this review were observational (*n* = 199, 94%). The limitations of observational research are well known, particularly the risk of bias [15]. However, the benefits of observational research are also acknowledged, such as feasibility for longer follow-up [16], which aligned with the results of this review (78% of studies involved intervention or follow-up for one year or more). While dialysis patients are sadly known to experience high mortality rates in general, appropriate time for follow up among this cohort remains relevant. For example, one study included in this review also tracked mortality and reported that following transtibial amputation, survival was 48% after approximately 2 years [17], identifying that long term outcomes are relevant for a large portion of dialysis patients who experience foot and lower limb health complications. Also, assessing bias was not a goal of this review, related to the extent and variability of included studies. However, assessing bias for existing experimental studies would be a beneficial next step in guiding future experimental research.

Only a small number of studies were identified which reported some of the outcomes of interest investigated in this review, including the management of foot and lower limb infection (*n* = 7, 3%) and associated hospital admission (*n* = 26, 12%). Furthermore, no studies were found to support evidence for interventions primarily related to exercise, offloading or education. As such, these may be areas for future research to guide foot and lower limb health management for dialysis patients. However, the overall health and capacity of dialysis patients must also be considered when planning interventions. Health and capacity factors of note may include the common occurrence of comorbidities, which one study reported impacting 82% of investigated ESRD patients in South Taiwan [18], and mental health challenges, which another study reported impacting 28% of ESRD patients in South Korea [19]. Also for consideration, given the high risk nature of foot and lower limb health for dialysis patients in combination with the body of evidence summarised in this review, future interventions should ideally be compared with standard care through randomised trials.

Studies identified in this review were conducted in various clinical settings. Clinical setting is important from a feasibility perspective as dialysis patients often struggle with severe fatigue and have to manage their available energy, juggling factors such as other medical appointments and travel time [20]. Although not captured in this review (related to this variable not being generally reported), the timing of intervention is also important for dialysis patients. Cognitive function for haemodialysis patients declines during dialysis, and is best immediately before or the day after dialysis [21]. Additionally, in practice we recognise that patients who attend dialysis units (hospital or satellite) are generally very eager to begin and finish dialysis promptly, which should be considered as it may impact feasibility if studies aim to work with patients during this time. Overall, clinical setting and timing are examples of variables which should be reported routinely in research regarding interventions for dialysis patients.

Of interest, 55% of the studies captured in this review (*n* = 117) included a population where dialysis patients made up less than 75% of the total study population. In many of these publications we noticed a trend where dialysis patients were mentioned only briefly in the abstract, hence full text review was required to determine eligibility, which led to high numbers of full text review (1032). Had these steps not been taken a large amount of evidence would have been missed. This should be considered when planning future related reviews and the importance of an appropriate search strategy is similarly emphasised [22].

Results from this review have limitations, some of which have already been acknowledged. For additional consideration, the broad inclusion criteria employed also at times made distinguishing whether studies met the inclusion criteria difficult and discussion between reviewers was employed. Also, no systematic search can capture all potentially relevant evidence. For example, more social support for dialysis patients is associated with less hospital admissions [23], however, this evidence was not captured in the current review as it has not been investigated specifically in relation to foot and lower limb health. Additionally, some dialysis patients are recognised as being more likely to experience health complications compared with others. For example, one study in Australia identified that Indigenous background was associated with amputation among dialysis patients (OR 3.39, 95% CI 1.38-8.33, p 0.01) [24]. However, analysis of subgroups was not an aim of the current review and hence none were conducted. Future investigation of distinct subgroups of dialysis patients should be approached collaboratively to support the achievement of goals which are meaningful to that group of dialysis patients, such as when working toward improved outcomes for Indigenous people [25].

## Conclusions

Foot and lower limb health complications are common, complex, and negative among dialysis patients. As such, a call for evidence to guide management is recurrent in the literature [1, 2, 5–7]. This review identified 212 such studies. Key findings included that studies most often aimed to both prevent and manage foot and lower limb health complications, the most common intervention type was surgery, studies frequently reported results for more than one foot or lower limb health outcome and, outcome results varied. These findings can serve as a platform in guiding future research, with a goal to support improved patient outcomes. Also, given the broad nature of foot and lower limb health complications for dialysis patients, interdisciplinary management may be indicated.

### Supplementary Information

**Additional file 1.** Search strategies.

**Additional file 2.** Extracted data.

## Data Availability

The dataset supporting the conclusions of this article is included within the article and its additional files (Additional file 2).

## Abbreviations

CINAHL: Cumulative index to nursing and allied health literature
OR: Odds ratio
PAD: Peripheral arterial disease
PRISMA-SR: Preferred reporting items for systematic reviews and meta-analyses-scoping review extension
